# Commercially Available Angiotensin II At_2_ Receptor Antibodies Are Nonspecific

**DOI:** 10.1371/journal.pone.0069234

**Published:** 2013-07-01

**Authors:** Roman Hafko, Sonia Villapol, Regina Nostramo, Aviva Symes, Esther L. Sabban, Tadashi Inagami, Juan M. Saavedra

**Affiliations:** 1 Section on Pharmacology, Division of Intramural Research Programs, National Institute of Mental Health, National Institutes of Health, Bethesda, Maryland, United States of America; 2 Centre for Neuroscience and Regenerative Medicine, Bethesda, Maryland, United States of America; 3 Department of Pharmacology, Uniformed Services University of the Health Sciences, Bethesda, Maryland, United States of America; 4 Department of Biochemistry and Molecular Biology, New York Medical College, Valhalla, New York, United States of America; 5 Vanderbilt University School of Medicine, Nashville, Tennessee, United States of America; Universtiy of Maryland Schoool of Medicine, United States of America

## Abstract

Commercially available angiotensin II AT_2_ receptor antibodies are widely employed for receptor localization and quantification, but they have not been adequately validated. In this study, we characterized three commercially available AT_2_ receptor antibodies: 2818-1 from Epitomics, sc-9040 from Santa Cruz Biotechnology, Inc., and AAR-012 from Alomone Labs. Using western blot analysis the immunostaining patterns observed were different for every antibody tested, and in most cases consisted of multiple immunoreactive bands. Identical immunoreactive patterns were present in wild-type and AT_2_ receptor knockout mice not expressing the target protein. In the mouse brain, immunocytochemical studies revealed very different cellular immunoreactivity for each antibody tested. While the 2818-1 antibody reacted only with endothelial cells in small parenchymal arteries, the sc-9040 antibody reacted only with ependymal cells lining the cerebral ventricles, and the AAR-012 antibody reacted only with multiple neuronal cell bodies in the cerebral cortex. Moreover, the immunoreactivities were identical in brain tissue from wild-type or AT_2_ receptor knockout mice. Furthermore, in both mice and rat tissue extracts, there was no correlation between the observed immunoreactivity and the presence or absence of AT_2_ receptor binding or gene expression. We conclude that none of these commercially available AT_2_ receptor antibodies tested met the criteria for specificity. In the absence of full antibody characterization, competitive radioligand binding and determination of mRNA expression remain the only reliable approaches to study AT_2_ receptor expression.

## Introduction

Circulating and local Renin-Angiotensin Systems (RAS) control multiple functions in many peripheral organs and in the brain [1–4]. The main active RAS component is Angiotensin II, which stimulates two major receptor types, AT_1_ and AT_2_ [1–3,5]. The AT_1_ receptors are considered the physiological Angiotensin II receptors; their signal transduction mechanisms and their role in the transmission of Angiotensin II effects have been firmly established [1–3,5]. AT_1_ receptor overactivity promotes peripheral vascular and tissue inflammation [6] and it is associated with essential hypertension, metabolic dysfunction, renal disease, brain inflammation and neuronal injury [4–7].

It has been proposed that AT_2_ receptor stimulation by Angiotensin II may normally counterbalance AT_1_ receptor activation, and that stimulation of AT_2_ receptors during AT_1_ receptor blockade is therapeutically beneficial [8]. AT_2_ receptor stimulation has been linked with activation of phosphatases leading to dephosphorylation of mitogen-activated protein (MAP) kinases, directly opposing MAP kinase activation through AT_1_ receptor stimulation [8]. Stimulation of AT_2_ receptors plays a protective role under pathological circumstances in the heart, kidney and brain, opposing AT_1_ receptor activation by increasing vasodilation and natriuresis and reducing brain ischemia and neuronal injury [8–12]. It appears that AT_2_ receptors contribute to control of AT_1_ receptor expression. In adult AT_2_ receptor knockout mice, AT_1_ receptor expression increases in the brain, adrenal gland, kidney, spleen and lung [13–16].

The possible beneficial effect of direct AT_2_ receptor stimulation has recently encouraged the development of novel AT_2_ receptor agonists, with the goal to protect peripheral organs and the brain from injury [15,16]. For these reasons the study of AT_2_ receptor function is generating increased interest. However, the role of the AT_2_ receptors has not been definitely clarified, and published results are controversial [13,17–21].

In support of a major role of AT_2_ receptors, antibodies have been used in hundreds of publications to determine receptor localization, quantification, immunoprecipitation, and other characteristics. For the most part, publications employed commercially available AT_2_ receptor antibodies. Unfortunately, the use of commercially available AT_2_ receptor antibodies results in variable, unpredictable, and above all, unreliable results.

To address this problem, we selected three commercially available antibodies raised against different domains of the AT_2_ receptor for characterization and comparative study. We used two polyclonal antibodies: sc-9040 from Santa Cruz and AAR-012 from Alomone, which had specific epitope sequences provided, and a monoclonal antibody 2818-1 from Epitomics, whose antigen sequence was stated to be within the C-terminal domain.

To characterize these antibodies, we followed established criteria [22–29]: 1) *The precise antigen sequence should be provided*; 2) *In western blots from tissues expressing AT*_*2*_ *receptors, the antibodies should detect immunoreactive bands of appropriate molecular weight*; 3) *Antibodies raised against different receptor domains must reveal similar patterns of immunoreactivity*; 4) *Antibody immunoreactivity should correlate with the degree of receptor expression as detected by additional methods such as competitive binding and/or mRNA expression*; 5) *Antibodies should not be reactive to tissues not expressing the target protein*; 6) *Immunocytochemistry must reveal similar tissue and cell localization for all antibodies.*

Availability of rodent tissues without AT_2_ receptor expression and a mouse AT_2_ receptor knockout strain [30–32] allowed a definitive proof of antibody specificity, a necessary condition to ensure that results are replicable and likely to be correct [22–29].

## Materials and Methods

### Animals

Male wild-type C57BL6/J were obtained from The Jackson Laboratory (Bar Harbor, MA). To produce the AT_2_^-/y^ receptor phenotype, mice (C57BL/6 and 129 Ola) were initially obtained from Jackson Laboratory (Bar Harbor, MA) and were subsequently housed at the Department of Biochemistry, Nashville University, where they were kept under controlled conditions, five per cage with free access to water and a standard diet at 22°C under a 12: 12 hour dark-light cycle. The AT_2_ receptor knockout mice were produced at the Department of Biochemistry, Nashville University as described previously [30]. Male mice were transported to NIMH when 8 weeks old and kept for one day under controlled conditions as above. The mice were sacrificed by cervical dislocation without prior anesthesia and their brainstems, containing the inferior olive, kidneys, spleens and adrenal glands were immediately dissected, snap-frozen in isopentane on dry ice and stored at -80°C until further processed.

Eight weeks old male Sprague Dawley rats were obtained from Charles River (Wilmington, MA, USA) and subsequently housed at the New York Medical College, two per cage with free access to water and a standard diet at 22°C under a 12: 12 hour dark-light cycle. The rats were sacrificed by fast decapitation and their adrenal glands, spleens, pituitary glands, livers, kidneys, cerebellums and ventro-posterior brain stems containing the inferior olive were immediately dissected, snap-frozen in liquid nitrogen and stored at -80°C until further processed.

The National Institute of Mental Health (Bethesda, MD, USA), (NIH Animal Care and Use Committee (protocol ASP SOP-03), and the New York Medical College Animal Care and Use Committee (protocol 84-1-0912H) approved all procedures. All efforts were made to minimize the number of animals used and their suffering (National Institutes of Health Guide for the Care and Use of Laboratory Animals, Publication No. 80-23, revised 1996).

### Western blot

Tissues from wild-type and AT_2_ receptor knockout mice and from rats were homogenized 1: 10 wt:vol in ice-cold RIPA buffer containing 50 mM Tris-HCl, 150 mM NaCl, 1% NP-40, 0.1% SDS, 0.5% sodium deoxycholate; supplemented with protease inhibitor cocktail (Roche Diagnostics, Indiana, IN) and centrifuged at 14,000xg for 10 min at 4°C. Protein concentration in the supernatant was determined by BCA assay (Thermo Scientific, Rockford, IL). The protein extracts were separated by SDS-PAGE using 12% NuPAGE Bis-Tris gels (Invitrogen) and transferred to PVDF membrane. The membranes were blocked in casein-based blocking buffer (Sigma-Aldrich) for 60 min at room temperature and exposed to primary antibodies overnight at 4°C. Rabbit antibody sc-9040 was purchased from Santa Cruz Biotechnology (Santa Cruz, CA, USA); 2818-1 from Epitomics, Inc. (Burlingame, CA, USA) and AAR-012 from Alomone Labs (Jerusalem, Israel). The information on immunogen used, specificity and applications, as provided by the manufacturers, is listed in Table 1. The antibody dilutions were as follows: sc-9040, 1:500; AAR-012, 1:200; 2818-1, 1:1000, as recommended by the manufacturers. After incubation, the membranes were washed and exposed to secondary horseradish peroxidase-conjugated donkey anti-rabbit antibody (GE Healthcare, Buckinghamshire, UK, catalog number NA934V, 1:5000) for 2 h at room temperature and then exposed to SuperSignal West Dura Substrate (Thermo Scientific) for chemiluminescent detection. After AT_2_ receptor detection, the membranes were stripped for 15 min at room temperature in Restore Western Blot stripping buffer (Thermo Scientific cat # 21059), blocked, exposed to β-Actin antibody (1:10000, Sigma-Aldrich, cat # A5441) for the mice tissues or β-Actin antibody (1:50000, Santa Cruz Biotechnology, cat # sc-47778) for rat tissues and chemiluminescence was detected as above.

**Table 1:** Characteristics of AT_2_ receptor antibodies used in the study.

**Antibody**	**Immunogen**	**Specificity**	**Application**
AT_2_, rabbit mAb, Epitomics, Inc., cat 2818-1	Non-specified peptide from C-terminus of human AT_2_ receptor	H, M, R	WB, IP
>AT_2_ (H-143), rabbit pAb, Santa Cruz Biotechnology, cat # sc-9040	Protein corresponding to amino acids 221-363 of AT_2_ of human origin	H, M, R	WB, IF, IP, ELISA
AT_2_, affinity purified rabbit pAb, Alomone Labs, cat # AAR-012	Peptide DNLNATGTNESAFNC corresponding to amino acid residues 21-35 of rat AT_2_ receptor	M, R	WB, IHC, ICC

mAb, monoclonal antibody; pAb, polyclonal antibody; H, human; M, mouse, R, rat; WB, Western blot; IHC, immunohistochemistry; ICC, immunocytochemistry; IF, immunofluorescence; IP, immunoprecipitation; ELISA, enzyme-linked immunosorbent assay

### RT-PCR

Total RNA was isolated from adrenal gland, kidney, spleen and brainstem of wild-type and AT_2_ receptor knockout mice, and from rat adrenal gland, spleen, kidney, liver, cerebellum, pituitary and inferior olive using TRIzol reagent (Invitrogen), followed by purification with RNeasy Mini kit (Qiagen, Valencia, CA, USA). Synthesis of complementary DNA (cDNA) was performed using 1 µg of total RNA and Super-Script III First-Strand Synthesis SuperMix for qRT-PCR (Invitrogen). Real-time PCR was performed in a 20 µl reaction mixture consisting of 10 µl SYBR Green PCR Master Mix (Applied Biosystems, Foster City, CA, USA), 4 µl cDNA and 0.3 µM of each primer for a specific target on a DNA Engine OpticonTM (Bio-Rad, Hercules, CA, USA). The primers used are listed in Table 2. Amplification was performed at 95 ^°^C for 15 min, followed by 45 cycles at 95 ^°^C for 20 s and 60 ^°^C for 60 s. Serial dilutions of mouse and rat adrenal medulla cDNA were used to obtain a standard curve. The relative amount of the target mRNA was normalized with the housekeeping gene GAPDH.

**Table 2:** List of PCR primers used in the study.

**Gene**	**Species**	**Accession #**	**Forward primer (5′–3′)**	**Reverse primer (5′–3′)**
AT_2_*	mouse	NM_007429.5	GGGTAAACAGACCCAGCAAA	CTGGAACTGTGCCCAGAAAT
AT_2_**	mouse	NM_007429.5	CATTGACCTGGCACTTCCTT	AAACACACTGCGGAGCTTCT
GAPDH	mouse	NM_008084.2	AACTTTGGCATTGTGGAAGG	GGATGCAGGGATGATGTTCT
AT_2_	rat	NM_012494.3	AACCGGCAGATAAGCATTTG	CAGCCACAGCCAGATTGAAG
GAPDH	rat	NM_017008.4	ATGACTCTACCCACGGCAAG	TGGAAGATGGTGATGGGTTT

AT_2_, Angiotensin II receptor type 2; GAPDH, Glyceraldehyde-3-phosphate dehydrogenase.

* AT_2_ primers targeting mRNA sequence corresponding to the non-disrupted part of AT2 receptor gene.

** AT_2_ primers targeting mRNA corresponding to the part of the gene in intron III that was deleted in AT2 receptor knockouts.

### Angiotensin II receptor binding

Freshly-frozen wild-type mouse adrenals and spleens and rat adrenals, spleens and kidneys were cut to 16 µm thick coronal sections in a cryostat at -20°C. The sections were thaw-mounted on gelatin-coated slides (Tekdon, Myakka City, FL), dried for 5 min at 50°C and stored at −80°C until used. Prior to use, the sections on slides were dried in a desiccator at room temperature and then lightly fixed in 0.2% formaldehyde (Mallinckrodt Chemicals, Philipsburg, NJ) in phosphate-buffered saline (PBS) for 10 min and washed twice for 5 min in PBS. The receptor autoradiography was performed as described previously [33]. The sections were preincubated for 15 min at 22°C in binding buffer containing 10 mM sodium phosphate buffer, pH 7.4, 0.005% bacitracin (Sigma-Aldrich, St Louis, MO), 5 mM Na _2_EDTA, 120 mM NaCl and 0.2% proteinase-free BSA (Sigma-Aldrich). Sections were then incubated for 2 h at 22°C in fresh binding buffer in the presence of 0.25 nM [^125^I] Sar^1^-Ile^8^-Angiotensin II (American Radiolabeled Chemicals, St. Louis, MO) to visualize total Angiotensin II binding to AT_1_ and AT_2_ receptors. To determine AT_2_ receptor-specific binding, consecutive sections were incubated as above in the presence of 10 µM of the AT_1_ receptor antagonist losartan (DuPont-Merck, Wilmington, DE) to displace binding to AT_1_ receptors. Slides were washed four times for 1 min in Tris-HCl, pH 7.4 at 4°C, followed by a 30 s rinse in distilled water at 4°C. Slides were then dried under a stream of cold air and exposed to Biomax MR film (Eastman Kodak, Rochester, NY). Images were analyzed by computerized densitometry using the Scion Image 4.0.2 for Windows (Scion Corporation, Frederick, Maryland, USA) based on the ImageJ Program of the National Institutes of Health [33].

### Immunohistochemistry and image acquisition

Wild-type and AT_2_ receptor knockout mouse 30µm thick brain coronal sections were cut in a cryostat at -16°C. Sections were post-fixed during 1 hour in paraformaldehyde (4% PFA) and were washed with 0.1% Triton-X100 in 0.1M phosphate buffered saline (PBST) and blocked in PBST/10% normal goat serum (NGS) for 1 hour, then incubated overnight at 4 ^o^C in PBST/1% NGS containing one of the three different rabbit anti-AT_2_ receptor antibodies; 2818-1 from Epitomics (dilution 1:400), sc-9040 from Santa Cruz (dilution 1:2000), and AAR-012 from Alomone (dilution 1:3000) together with the mouse monoclonal anti-NeuN antibody from Millipore (dilution 1:200). Sections were then washed in PBST three times for a period of 10 minutes and incubated against the AT_2_ receptor antisera with Alexa Fluor 568-conjugated IgG secondary antibody (1:1000; Jackson Immunoresearch) and against the neuronal marker NeuN with Alexa Fluor 488-conjugated IgG secondary antibody (dilution 1:1000; Jackson Immunoresearch) for 2 hours at room temperature. Sections were rinsed once with PBST followed by a distilled water rinse before coverslipping with ProLong Gold antifade reagent (Invitrogen). Images were acquired at 40x magnification on an Olympus BX61 with attached qImaging Retiga EXi Aqua CCD camera, and iVision software (BioVision Technologies, Exton, PA) and cropped and adjusted using Adobe Photoshop CS5.

## Results

### Angiotensin II receptor mRNA expression and AT_2_ receptor binding in wild-type and AT_2_ knockout mice

**Figure 1:**
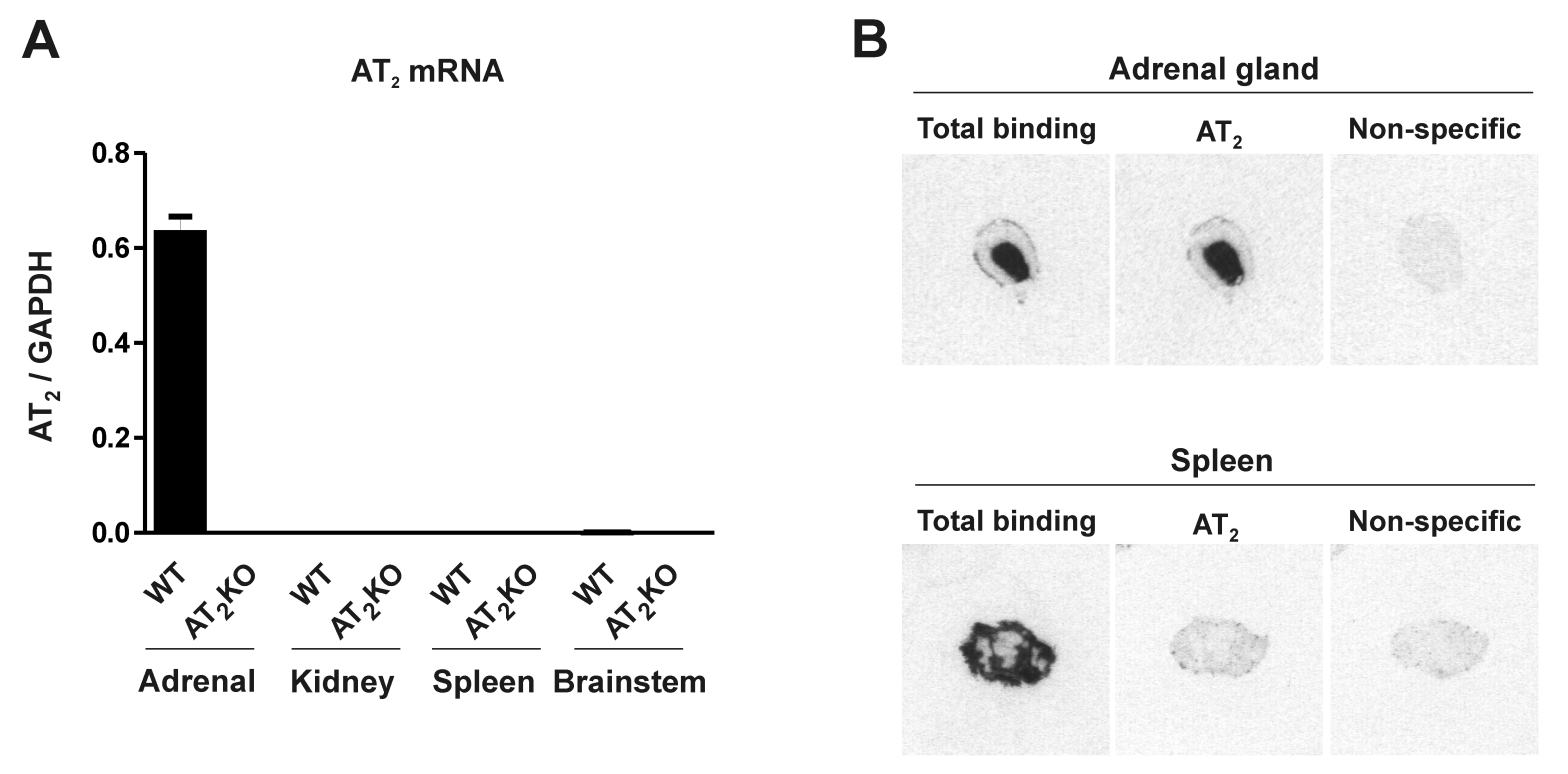
Angiotensin II AT_2_ receptor mRNA and AT_2_ receptor binding in wild-type and AT_2_ knockout mice. (A) Expression of mRNAs was studied by RT-PCR in adrenal gland, kidney cortex, spleen and brainstem of wild-type and AT_2_ knockout mice. Gene expression was normalized to the level of GAPDH, and represents the mean ± S.E.M. of three individual determinations. Note that AT_2_ mRNA is only expressed in adrenal glands of wild-type mice, and not expressed in kidney cortex, spleen and brainstem of wild type or AT_2_ receptor knockout mice. (B) AT_2_ receptor expression was studied by quantitative autoradiography in sections from adrenal gland and spleen, incubated in the presence of 0.25 nM [^125^I] Sar^1^-Ile^8^-Angiotensin II as described in Materials and Methods. Figures represent a typical result repeated in three different wild-type mice. Note abundant receptor binding in the adrenal medulla and spleen, and lower expression in the adrenal cortex. Binding was partially displaced in the adrenal cortex, and completely displaced in the spleen, by incubation with the AT_1_ receptor-specific antagonist losartan. Non-specific binding was the result of incubation in the presence of non-radiolabeled Angiotensin II. The results indicate that most of the receptor binding in the adrenal medulla, and part of the binding in the adrenal cortex is insensitive to losartan expression, revealing the expression of AT_2_ receptors. Conversely, binding in the spleen was completely displaced by losartan, indicating that spleen receptors are exclusively of the AT_1_ subtype.

AT_2_ receptor mRNA was clearly expressed in the adrenal glands but was undetectable in the kidney, spleen and brain stem of wild-type mice. In AT_2_ receptor knockout mice, it was absent from all tissues studied (Figure 1A). Identical results were obtained whether PCR primers were targeting mRNA sequence corresponding to the non-disrupted part of AT_2_ receptor gene (Figure 1A) or the sequence corresponding to the part of the gene in intron III that was deleted in AT_2_ receptor knockouts (data not shown) (Table 2).

Wild-type mice expressed Angiotensin II receptor binding in the adrenal zona glomerulosa and medulla, and in the spleen (Figure 1B). Incubation with the AT_1_ receptor selective ligand losartan partially displaced Angiotensin II binding from the adrenal zona glomerulosa, and completely displaced all binding from the spleen (Figure 1B). This indicated that AT_2_ receptor binding was highly expressed in the adrenal medulla, was also expressed in the adrenal zona glomerulosa, and was absent from the spleen (Figure 1B).

### AT_2_ receptor protein expression in adrenal, spleen, kidney and brainstem of wild-type and AT_2_ knockout mice

**Figure 2:**
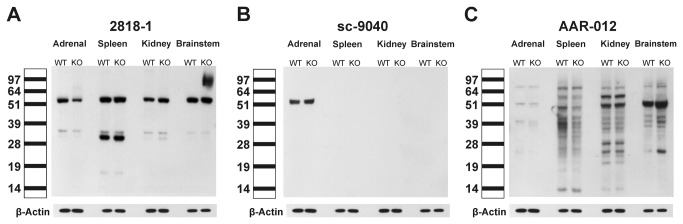
AT_2_ receptor protein expression in adrenal, spleen, kidney and brainstem of wild-type and AT_2_ knockout mice. Angiotensin II AT_2_ receptor protein expression was studied by western blotting. Protein extracts were separated by SDS-PAGE electrophoresis and exposed to three different anti-AT_2_ receptor antibodies as indicated by catalog number at the top of the picture. The scale on the left indicates the size in kDa according to the positions of the protein ladder. The reported size of the AT_2_ receptor is about 50-55 kDa. Note that each antibody tested generated different immunoreactivity patterns, that these patterns do not correlate with the presence or absence of the target protein, and that in all cases there is no difference between band intensity obtained from tissues from AT_2_ knockout and wild-type mice. The figure represents a typical experiment repeated two times in individual samples.

The AT_2_ receptor antibodies sc-9040, AAR-012 and 2818-1 were tested by Western blot using adrenal, spleen, kidney and brainstem protein extracts from wild-type and AT_2_ receptor knockout mice. In all cases, the immunoreactivity of extracts of tissues from wild-type mice was not different from extracts of tissues from AT_2_ receptor knockout mice (Figure 2A, 2B and 2C). This demonstrated that the pattern and intensity of all bands detected was similar whether the target protein was present or not. Furthermore, the pattern and the intensity of the immunoreactivity detected by each antibody and in every tissue studied were very different, indicating that the immunoreactivity depended on the antibody tested and not the presence or absence of the target protein (Figure 2A, 2B and 2C).

All tested antibodies revealed bands of approximately 50-55 kDa. Antibody 2818-1 yielded 50-55 kDa bands in all tissues studied, and the intensity of the bands obtained from spleen, kidney and brainstem were of higher intensity than those obtained from adrenal extracts (Figure 2A). Furthermore, antibody 2818-1 yielded 30-35 kDa immunoreactivity bands, particularly intense in spleen extracts from both wild-type and AT_2_ receptor knockout mice, and the brainstem of AT_2_ receptor knockout mice showed major immunoreactivity in the 70-97 kDa region (Figure 2A).

Antibody sc-9040 yielded a single band, of similar intensity, at the 50-55 kDa range for adrenal gland extracts of both wild-type and AT_2_ receptor knockout mice, and did not react with spleen, kidney or brainstem extracts (Figure 2B).

Antibody AAR-012 revealed multiple bands between 60 and 14 kDa, predominant in spleen, kidney and brainstem extracts but very weak in adrenal extracts (Figure 2C). Most of the bands were of identical intensity in wild-type and knockout mice (Figure 2C).

### Immunohistochemistry of AT_2_ receptor antibodies in mouse brain

**Figure 3:**
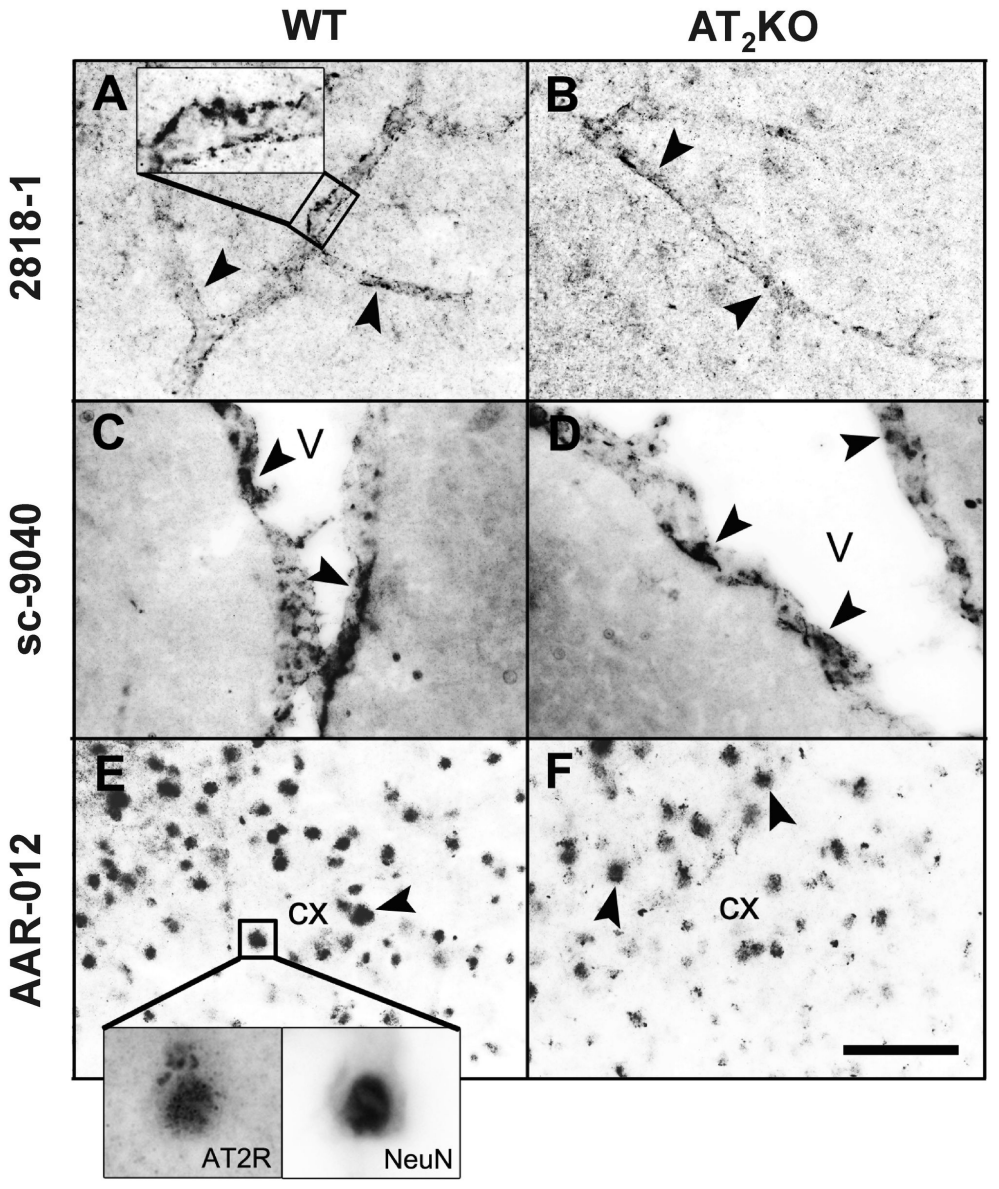
Angiotensin II AT_2_ receptor immunocytochemistry in the mouse brain. (A) and (B) Antibody 2818-1 from Epitomics (dilution 1:400). The AT_2_ receptor antibody reacts with parenchymal microvessels (indicated with arrowheads in A and B, and in box in panel A) in the cerebral motor cortex from -1.22 mm to Bregma, and the immunocytochemistry is similar in wild-type (WT) (A) and AT_2_ knock-out (KO) mice (B). (C) and (D) Antibody sc-9040 from Santa Cruz (dilution 1:2000). The antibody detects ciliated ependymal cells (arrowheads) of the lateral ventricle (V), and the staining is similar in the wild type (C) or knockout mice (D) located at the same coordinates as A and B. (E) and (F): antibody AAR-012 from Alomone (dilution 1:3000). In the cerebral motor cortex, the antibody detects cell colocalization with the neuronal marker NeuN (indicated with arrowheads in E and F and in the boxes in panel E). The staining is similar in wild-type (E) and AT_2_ receptor knockout (F) mice. Scale bar = 20 µm.

All antibodies tested revealed selective immunocytochemical profiles when tested in the mouse brain. However, these profiles were strikingly different for each antibody tested, and the profiles were identical for wild-type (Figure 3A, 3C and 3E) and AT_2_ receptor knockout (Figure 3B, 3D and 3F) mice. Negative controls were performed using mouse (red) and rabbit (green) IgG with no primary antibody.

The 2818-1 antibody reacted with parenchymal microvessels in the cerebral cortex, with specific staining restricted to the endothelial cells (Figure 3A and 3B). The sc-9040 antibody reacted selectively with ciliated ependymal cells bordering the lateral ventricle (Figure 3C and 3D). The AAR-012 antibody stained numerous cells in the cerebral cortex, predominantly neurons (NeuN positive cells), with more pronounced nuclear staining, and lesser staining in the cytoplasm (Figure 3E and 3F).

### Angiotensin II receptor mRNA expression and AT_2_ receptor binding in rat tissues

**Figure 4:**
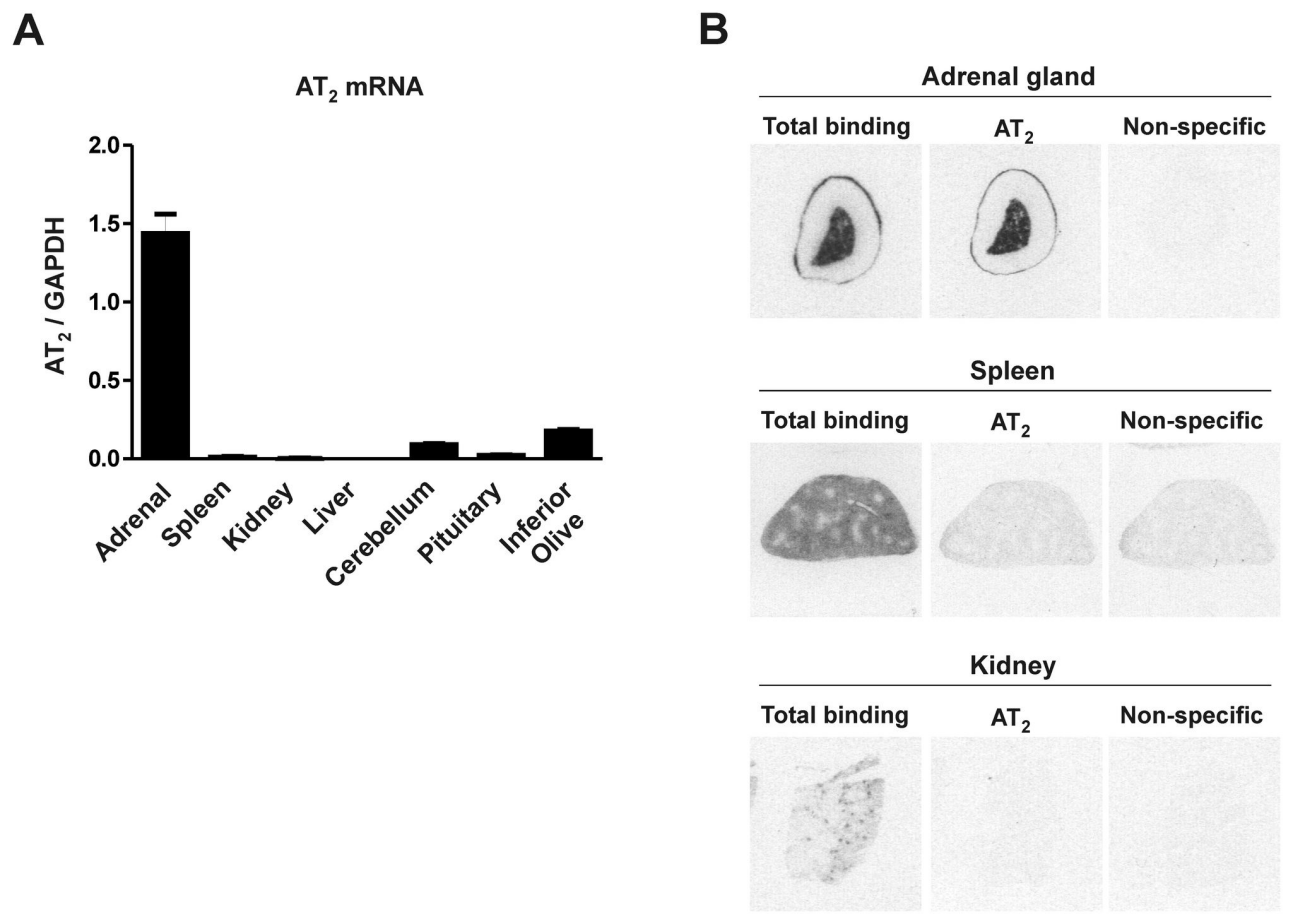
Angiotensin II AT_2_ receptor mRNA and AT_2_ receptor binding in rat tissues. (A) Expression of mRNAs was studied by RT-PCR in adrenal gland, spleen, kidney cortex, liver, cerebellum, pituitary and inferior olive of adult male Sprague Dawley rats. Gene expression was normalized to the level of GAPDH, and represents the mean ± S.E.M. of three individual determinations. Note that AT_2_ mRNA is only expressed in adrenal glands, inferior olive and cerebellum, and it is not detectable in the spleen, kidney, liver and pituitary. (B) AT_2_ receptor expression was studied by quantitative autoradiography in sections from adrenal gland and spleen, incubated in the presence of 0.25 nM [^125^I] Sar^1^-Ile^8^-Angiotensin II as described in Materials and Methods. Figures represent a typical result repeated in three different rats. Note abundant receptor binding in the adrenal medulla and spleen, and lower expression in adrenal zona glomerulosa and kidney cortex. In the kidney cortex, binding was localized to the glomeruli. Binding was partially displaced in the adrenal cortex, and completely displaced in the spleen and kidney cortex, by incubation with the AT_1_ receptor-specific antagonist losartan. Non-specific binding was the result of incubation in the presence of non-radiolabeled Angiotensin II. The results indicate that most of the receptor binding in the adrenal medulla, and part of the binding in the adrenal cortex is insensitive to losartan expression, revealing the expression of AT_2_ receptors. Conversely, binding in the spleen and kidney cortex was completely displaced by losartan, indicating that spleen and kidney cortex receptors are exclusively of the AT_1_ subtype (B).

AT_2_ mRNA expression was determined by qRT-PCR in rat tissues normally expressing (adrenal medulla, cerebellum, inferior olive) or not expressing (spleen, kidney, liver, pituitary) AT_2_ receptors. As expected, clear mRNA expression was found in the adrenal medulla, and lesser expression was detected in the inferior olive and cerebellum (Figure 4A). AT_2_ mRNA expression in the spleen, kidney, liver and pituitary were below the level of detection of our experiments (Figure 4A).

Rats expressed Angiotensin II receptor binding in the adrenal zona glomerulosa and medulla, the spleen and the kidney cortex (Figure 4B). Incubation with the AT_1_ receptor selective ligand losartan partially displaced Angiotensin II binding from the adrenal zona glomerulosa, and completely displaced all binding from the spleen and kidney cortex (Figure 4B). This indicated that AT_2_ receptor binding in the rat was highly expressed in the adrenal medulla, was also expressed in the adrenal zona glomerulosa, and was absent from the spleen and kidney (Figure 4B).

### AT_2_ receptor protein expression in rat tissues

The AT_2_ receptor antibodies sc-9040, AAR-012 and 2818-1 were tested by Western blot using protein extracts from rat adrenal, spleen, kidney, liver, cerebellum, pituitary and inferior olive.

**Figure 5:**
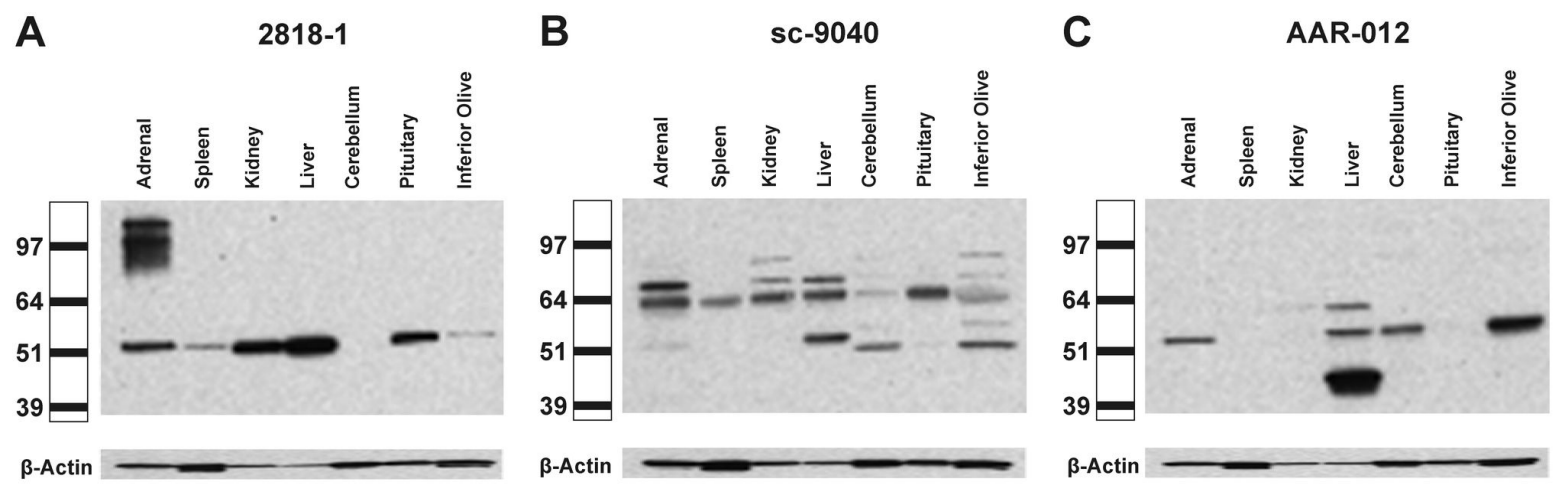
AT_2_ receptor protein expression in rat tissues. Angiotensin II AT_2_ receptor protein expression was studied by western blotting in adult male Sprague Dawley rat tissues expressing (adrenal gland, cerebellum, inferior olive) and not expressing (spleen, kidney, liver, pituitary) AT_2_ receptor mRNA or binding. Protein extracts were separated by SDS-PAGE electrophoresis and exposed to three different anti-AT_2_ receptor antibodies as indicated by catalog number at the top of the picture. The scale on the left indicates the size in kDa according to the positions of the protein ladder. The reported size of the AT_2_ receptor is about 50-55 kDa. Note that each antibody tested generated different immunoreactivity patterns that these patterns do not correlate with the presence or absence of the target protein. The figure represents a typical experiment repeated two times in individual samples.

The pattern and the intensity of the immunoreactivity detected by each antibody and in every tissue studied were very different (Figure 5A, 5B and 5C). Immunoreactivity was detected, for all antibodies tested, in rat tissues expressing or not expressing AT_2_ receptors. In no case did such immunoreactivity correlate with the reported presence or absence of AT_2_ receptor mRNA or receptor binding (Figure 5A, 5B and 5C). In addition, immunoreactivity detected in rat adrenal, spleen and kidney was very different, for all antibodies tested, from the immunoreactive patterns observed in mouse tissues (Figure 5A, 5B and 5C compare with Figure 2A, 2B and 2C). This indicated that the immunoreactivity depended on the antibody tested and the rodent species studied, and not the presence or absence of the target protein (Figure 5A, 5B and 5C).

Antibody 2818-1 yielded strikingly different 50-55 kDa bands in all tissues studied except the cerebellum (Figure 5A). The intensity of the bands in the kidney and liver were clearly higher than that in the adrenal (Figure 5A). In addition adrenal extracts expressed multiple prominent immunoreactive bands that were observed in the 80-110 kDa region (Figure 5A).

Antibody sc-9040 yielded several immunoreactive bands in the 64-85 kDa region, and these bands were of very different intensity in each of the tissues studied (Figure 5B). Immunoreactive bands of different intensity were also observed in the 50-55 kDa region of the liver, cerebellum, inferior olive and adrenal (Figure 5B).

Antibody AAR-012 revealed immunoreactive bands in the 50-55 kDa region of the adrenal, liver, cerebellum and inferior olive (Figure 5C). In the liver, there was an additional immunoreactive band in the 60 kDa region, and intense immunoreactivity in the 40-45 kDa region (Figure 5C).

## Discussion

This study shows that each of the three commercially available AT_2_ receptor antibodies tested yields markedly different immunoreactivity patterns and immunocytochemical reactivity. For each antibody, the immunoreactivity differs with the tissue and the rodent species studied and does not correlate with the expression of the AT_2_ receptor protein. The results demonstrate that each of these antibodies reacts with multiple off-target proteins, and that the immunoreactivity observed is not related to the presence or absence of the target protein.

A number of antibodies, most of them commercially available, have been long used for studying the localization, expression and regulation of AT_2_ receptors. However, the use of some of these antibodies in our laboratory invariably revealed a lack of specificity. The goal of the study was to determine whether or not commercially available AT_2_ receptor antibodies were specific, and consequently, whether results obtained with their use may generate reliable results.

A preliminary survey identified 10 commercially available AT_2_ receptor antibodies (Tables 1 and 3). Many of the antibodies did not provide either the epitope sequence or validating experimental data (Table 3). Three antibodies providing information on their epitope sequences were selected for our study.

**Table 3:** Additional available AT_2_ receptor antibodies not tested.

**Antibody**	**Immunogen**	**Specificity**	**Application**
AT_2_ (C-18), affinity purified goat pAb Santa Cruz Biotechnology, cat # sc-7420>	Non-specified peptide from C-terminal cytoplasmic domain of AT_2_ of human origin	H, M, R	WB, IF, IP, ELISA
AT_2_ (C-15), affinity purified goat pAb Santa Cruz Biotechnology, cat # sc-48451	Non-specified peptide from C-terminal cytoplasmic domain of AT_2_ of human origin	H, M, R (E, C, B, P)	WB, IF, ELISA
AT_2_ (K-15), affinity purified goat pAb Santa Cruz Biotechnology, cat # sc-48452	Non-specified peptide from extracellular domain of AT_2_ of human origin	H, M, R (E, C, B, P)	WB, IF, IHC, ELISA
AT_2_, rabbit mAb, Abcam, cat # Ab92445	Non-specified peptide from C-terminus of human AT_2_ receptor	H, M, R	WB, IP
AT_2_ affinity purified rabbit pAb Milipore, cat # AB15554	Non-specified peptide from C-terminus of rat AT_2_ receptor	M, R	WB, IC
AT_2_, rabbit pAb, LifeSpan BioSciences, cat # LS-C11856	Non-specified peptide close to C-terminus of human AT_2_ receptor	H, M, R	WB, ELISA
AT_2_, rabbit pAb, Novus Biologicals, cat # NBP1-77368>	Non-specified peptide near to the center of human AT_2_ receptor	H, M, R	WB, IHC, ELISA

mAb, monoclonal antibody; pAb, polyclonal antibody; H, human; M, mouse, R, rat; E, equine; C, canine; B, bovine; P, porcine; WB, Western blot; IHC, immunohistochemistry; ICC, immunocytochemistry; IF, immunofluorescence; IP, immunoprecipitation; ELISA, enzyme-linked immunosorbent assay

To test the validity of the antibodies, we applied several characterization criteria, as established earlier [22–24]. The criteria selected were as follows:

1. *The precise antigen sequence should be provided.*

Two of the AT_2_ receptor antibodies studied, sc-9040 and AAR-012, raised against different extracellular domains, met this criterion (Table 1). The 2818-1 monoclonal antibody was provided without the sequence of the immunizing peptide but with a statement that it was raised against part of the C-terminal domain. Seven additional AT_2_ antibodies failed to provide the antigen sequence (Table 3). For this reason these seven antibodies were not considered for the study.

2. *The antibodies must detect, in western blots from tissues expressing AT_2_ receptors, single bands of appropriate molecular weight or additional bands, again of appropriate molecular weight, if the antigen has several known molecular configurations.*

Molecular weights of the recognized proteins reported by commercially available antibody sources were not uniform. Information provided by commercial sources on all three antibodies selected for this study provided immunoreactive bands of different molecular weights, ranging from about 45 to 50 kDa (Table 1). Information on other commercially available antibodies, not studied here, reported reactive proteins of variable molecular weight, ranging from 36 to 50 kDa (Table 3). No additional immunoreactive bands have been reported in the Product Data sheets for any of the commercially available antibodies.

The reported molecular weights for the AT_2_ receptors varied from 30 to 120 kDa depending on the conditions of the study, the organ considered, the presence of homodimers and trimers, and the degree of glycosylation [34–37]. Overall, molecular weights from 68 to 113 kDa had been the most frequent findings [34–37]. Our study revealed, in many but not all tissues tested, immunoreactive bands of molecular weights not corresponding to those reported in the literature. In many cases antibodies reacted with multiple proteins of variable molecular weight, higher or lower than 50-55 kDa, ranging from about 130 to 14 kDa, and some of these bands were very predominant. Moreover, western blots revealed that the immunoreactive patterns for each antibody tested were strikingly different and variable according to the tissue examined and the rodent species studied.

3: *Antibodies raised against different antigen domains must reveal similar patterns of immunoreactivity.*

As detected in western blots, immunoreactive patterns were strikingly different for each of the antibodies tested. The patterns varied with the antibody, the tissue and the rodent species studied. These findings clearly indicate that each antibody recognized different sets of immunoreactive proteins, and that the immunoreactivity was tissue and species-dependent for each antibody studied.

4. *Antibody immunoreactivity should correlate with the degree of receptor expression as detected by additional methods such as competitive binding and/or mRNA expression. Antibodies should not be reactive to tissues not expressing the target protein.*

We tested each antibody for immunoreactivity in wild-type and AT_2_ receptor knockout mice and in mouse and rat tissues expressing (adrenal gland, inferior olive) and not expressing (liver, spleen, kidney, pituitary gland) AT_2_ receptors, as determined by qRT-PCR, *in situ* hybridization and receptor binding, in the present experiments and in the literature [10,33,38–54].

We found that the immunoreactivity of the antibodies tested did not correlate with the reported expression of the AT_2_ receptor binding or mRNA. One example is the mouse and rat kidney. While the mouse kidney expresses low levels of AT_2_ receptors [12,39,55] we could not detect AT_2_ receptor mRNA or AT_2_ receptor binding in the kidney of the male rat, as reported earlier [38,56,57] Conversely, AT_2_ receptors have been localized to kidneys of male rats with the use of AT_2_ receptor antibodies [8].

As determined in western blots, each antibody revealed identical immunoreactivity bands, at about 50-55 kDa, in wild-type and AT_2_ receptor knockout mice. Furthermore multiple additional and major immunoreactive bands were observed in wild-type and AT_2_ receptor knockout mice tissues, and in mouse and rat tissues where the AT_2_ receptor mRNA and binding could not be detected (present results and [10,33,38–41,46–54,58]). Moreover, each antibody tested revealed diverse immunoreactive patterns, and immunoreactivity was different for each tissue examined and when similar tissues from mice and rats were compared. The conclusion is that the reported immunoreactivities do not correspond to the presence or absence of the target protein.

### Expression of non-deleted domains in AT_2_ receptor knockout mice

In AT_2_ knockout mice, the AT_2_ receptor was disrupted by insertion of a neomycin resistance cassette resulting in a deletion of about 5kb of the coding region [30,59], and corresponding to amino acids 1-142 of the mouse AT_2_ receptor. One of the antibodies tested (AAR-012 from Alomone lab) was raised against amino acids 21-35, untranslated in AT_2_ knockout mice. The other two antibodies studied here were raised against amino acid sequences corresponding to domains not deleted in the AT_2_ gene. AT_2_ antibody sc-9040 from Santa Cruz Biotechnology was raised against amino acids 221-363. A synthetic peptide corresponding to residues at the C-term of AT_2_ receptor was used as an immunogen for the preparation of the 2818-1 antibody from Epitomics, Inc., but the peptide sequence was not provided. While it is possible that the non-functional fragment of the protein potentially expressed may interact with the sc-9040 or the 2818-1 antibodies, the potential immunoreactive fragments would show molecular sizes different from the wild type AT_2_ receptor. Instead, each of the antibodies tested yielded absolutely identical bands when the receptor is present, as in the wild-type mice, whether a major part is deleted or truncated, as in the knockout mice, or when the AT_2_ gene is not present, as in the rat liver, kidney or pituitary gland or mouse spleen and liver (present results and [10,33,38–41,46–54]. This indicates that bands observed in knockout mice do not reflect AT_2_ receptor protein fragments but additional off-target proteins unrelated to the AT_2_ receptor.

5. *Immunocytochemistry must reveal similar tissue and cell localization for all fully characterized antibodies*. Our study revealed surprisingly specific cellular staining in the mouse brain. However, each antibody tested selectively stained different cells, and in all cases immunocytochemistry was identical in cells from wild-type and AT_2_ receptor knockout mice. Therefore, in all cases the results depended on the antibody tested and not on the presence of AT_2_ receptors.

In keeping with the present observations, recent publications reported the lack of specificity of commercially available and newly developed non-commercial AT_1_ receptor antibodies [28,29,60,61]. Moreover, previous reports questioned the specificity of a large number of G-protein-coupled receptor antibodies, including muscarinic, adrenergic, galanin, and dopamine receptors [27,62–67]. These reports indicate that, unfortunately, lack of specificity is the norm rather than the exception for GPCR antibodies.

In conclusion, the antibodies considered here do not meet any of the established and necessary characterization criteria, and they are not specific for the AT_2_ receptor. This study demonstrates the need for caution in interpreting the results of experiments using AT_2_ receptor antibodies that are not strictly characterized.
