# Correction: Commercially Available Angiotensin II AT_2_ Receptor Antibodies Are Nonspecific

**DOI:** 10.1371/annotation/beee9a88-80e1-4332-ac02-c1845beaa71b

**Published:** 2014-01-07

**Authors:** Roman Hafko, Sonia Villapol, Regina Nostramo, Aviva Symes, Esther L. Sabban, Tadashi Inagami, Juan M. Saavedra

An error was introduced in the preparation of this article for publication. The word "At_2_" is incorrect in the article title. The correct title is 

"Commercially Available Angiotensin II AT_2_ Receptor Antibodies Are Nonspecific". 

The correct citation is: 

Hafko R, Villapol S, Nostramo R, Symes A, Sabban EL, et al. (2013) Commercially Available Angiotensin II AT_2_ Receptor Antibodies Are Nonspecific. PLoS ONE 8(7): e69234. doi:10.1371/journal.pone.0069234

